# Evénements indésirables peropératoire: lecture critique du registre du bloc opératoire de l’hôpital militaire Moulay Ismail Meknès

**DOI:** 10.11604/pamj.2016.24.178.7648

**Published:** 2016-06-30

**Authors:** Hicham Kechna, Omar Ouzzad, Khalid Chkoura, Jaouad Loutid, Moulay Ahmed Hachimi, Sidi Mohamed Hanafi

**Affiliations:** 1 Service d’Anesthésiologie, Hôpital Moulay Ismail, Meknès, Maroc; 2 Service de Réanimation, Hôpital Moulay Ismail, Meknès, Maroc; 3 Service des Urgences, Pôle d’Anesthésie Réanimation et Urgence, Hôpital Moulay Ismail, Meknès, Faculté de Médecine et de Pharmacie, Université Sidi Mohamed Ben Abdellah, Fès, Maroc

**Keywords:** Incidents, accidents anesthésiques, complications au bloc opératoire, Incidents, anesthetic accidents, complications in the Surgery Depatment

## Abstract

**Introduction:**

Malgré les importants progrès qui ont été faits dans le domaine de la sécurité en anesthésie, la morbidité (grave ou non, liée complètement ou partiellement à l’anesthésie) reste cependant fréquente, et aucun praticien n’est aujourd’hui à l’abri d’un accident. Dans le contexte actuel où la priorité est donnée à la formation, à l’amélioration de la qualité et de la sécurité des soins, la survenue d’un accident d’anesthésie au bloc opératoire est un événement extrêmement traumatisant. La crainte de poursuite, le contexte émotionnel rendent cette gestion parfois très difficile. Pour cette raison, elle doit faire l’objet d’une codification, à la manière des protocoles de bloc, avec trois grands axes de gestion: le patient victime, le personnel médical et paramédical impliqué et l’analyse de l’incident pour éviter une récidive.

**Méthodes:**

Dans un but d’améliorer les soins prodigués au bloc opératoire nous avons établi un registre où sont consignés continuellement les différents incidents et accidents survenu soit en salle opératoire ou en salle de surveillance post interventionnelle. Une première lecture a été faite à l’occasion des Journées d'Enseignement Post Universitaire (JEPU) de Fès (Maroc) organisées en partenariat avec les JEPU de la Pitié salpêtrière de Paris à la faculté de Médecine et de Pharmacie de Fès sous le thème: «Les Situations Critiques Au Bloc Opératoire» les 17 et 18 Avril 2015.

**Résultats:**

1761 patients ont été admis aux différentes salles du bloc opératoire dont 96 en salle d’endoscopie et 17 sédations en radiologie. 29 patients (1.64%) ont présentés un incident et/ou un accident en péri opératoire. La plupart des effets indésirables sont survenus en per opératoire (58,6%). Dans 28,6% des cas en postopératoire immédiat ou en salle de surveillance post interventionnelle (SSPI). La plupart des complications survenues sont d’ordre respiratoire (34%) ou cardio vasculaire (31%). On a colligé 5 décès en périopératoire soit une mortalité de 0,28%. La détermination de la cause n’est pas toujours évidente. Le facteur humain serait responsable de 24% des incidents.

**Conclusion:**

Cette observation illustre les différents événements indésirables survenus depuis la création de ce registre il y a 6 mois. Nous proposons une lecture critique de ce registre dans le seul souci est d’améliorer nos pratiques dans une perspective de renforcer la sécurité anesthésique.

## Abstract (en)

**Introduction:**

Despite significant progress made in the field of safety in anesthesia, morbidity (serious or not, completely or partially related to anesthesia) remains common and no health practitioner is immune from accidents. In the current context where priority is given to training programmes, to quality and safety improvement in health care, the occurrence of an anesthesia-related accident in the surgery department is an extremely traumatic event. The fear of prosecution, the emotional context make it difficult to manage. For this reason, it must be codified according to Department protocols, based on three main axes of management: patient victim, medical and paramedical staff involved and accident analysis to prevent its recurrence.

**Method:**

In order to improve health care quality in surgery department we have implemented a continuously updated data registry containing the incidents and accidents occurred either in the operating room or in the post-interventional surveillance room A first reading was made on the occasion of Post Doctoral Training (JEPU) in Fez (Morocco) organized in partnership with the JEPU of the Pitié Salpêtrière in Paris at the School of Medicine and Pharmacy of Fez under the theme: «Critical Situations In Surgery Department» 17,18 April 2015.

**Results:**

1761 patients were admitted to different operating rooms, 96 in the endoscopy room and 17 under sedation in diagnostic radiology. 29 patients (1.64%) reported a perioperative incident and/or a perioperative accident. Most of the adverse events occurred during surgery (58.6%). 28.6% of cases in the immediate post-operative period or in the post-interventional surveillance room. The most frequent complications were respiratory (34%) or cardiovascular (31%). We recorded 5 perioperative deaths (mortality rate of 28%) Determining the cause is not always obvious. The human factor is responsible for 24% of incidents.

**Conclusion:**

This observation highlights the different adverse events which have been recorded since the establishment of the data registry 6 months before. We offer a critical reading of this data registry for the sole purpose of improving our practices with a view to strengthening safety during anaesthesia.

## Introduction

Malgré les importants progrès qui ont été faits dans le domaine de la sécurité en anesthésie, la morbidité reste cependant fréquente, et aucun praticien n’est aujourd’hui à l’abri d’un accident. Dans un but d’améliorer les soins prodigués au bloc opératoire et en réponse aux objectifs d’évaluation des pratiques professionnelles maintenant obligatoire [1], nous avons établi un registre où sont consignés continuellement les différents incidents et accidents survenus soit en salle opératoire ou en salle de surveillance post interventionnelle. Nous proposons une lecture critique de ce registre dans le seul souci d’améliorer nos pratiques dans une perspective de renforcer la sécurité anesthésique.

## Méthodes

Notre étude, qui est une étude transversale descriptive, étalée sur une durée de 7 mois du 1^er^ septembre 2014 jusqu’au 12/04 /2015, aura pour objectif d’évaluer l’incidence des effets indésirables survenus en péri opératoire, la nature de ces complications et de rechercher leurs facteurs favorisants afin de pouvoir les prévenir. Elle devra à terme (un an) déboucher sur un certain nombre de recommandations visant à améliorer la pratique de l’anesthésie et l’amélioration de la sécurité des patients au sein de notre établissement. Un registre de recueil de l’activité, des techniques et des incidents anesthésiques a été instauré dans le bloc opératoire de l’hôpital militaire My Ismail à Meknès. Les données sont remplies manuellement pour chaque patient opéré ayant présenté un événement indésirable quel que soit sa nature avec description détaillée des techniques d’anesthésie et des incidents anesthésiques. L’analyse a porté sur les données recueillies concernant tous les patients opérés au bloc opératoire central avec 8 salles et les actes nécessitant une sédation notamment en endoscopie et en radiologie. Ont été exclus les patients opérés aux urgences. Sur chaque page du registre ont été consigné l’identité du patient, ses antécédents, score ASA, le type d’anesthésie et sa durée, les produits anesthésiques utilisés, les incidents et les accidents per opératoires, la nature des accidents postopératoires et leur évolution; ainsi que le séjour en SSPI et le lieu de transfert des patients. Le recueil des accidents et/ou incidents se fait sous la responsabilité des médecins anesthésistes du pôle. Certaines complications (la douleur postopératoire, les l’hypothermie, les frissons post opératoires et les nausées vomissements post opératoires…) ne sont pas mis en exergue pour cette première illustration car elles engagent en grande partie la disponibilité des moyens préventifs recommandés par les sociétés savantes.

## Résultats

1761 patients ont été admis aux différentes salles du bloc opératoire dont 96 en salle d’endoscopie et 17 sédations en radiologie; la majorité des patients est de sexe masculin avec un taux de 63.36%; l’âge moyen de nos patients est de 48 ans avec des extrêmes allant de 1 an à 82 ans; l’anesthésie générale a été pratiquée chez 40%, l’anesthésie péri médullaire chez 29%, l’anesthésie locorégionale chez 6% et l’anesthésie locale chez 25% des patients; le pourcentage des patients opérés par spécialité et par ordre décroissant est le suivant: l’ophtalmologie 24%, la traumatologie 17%, l’urologie et la viscérale (chacune 13 %), l’ORL 12%, la gynécologie, la vasculaire et la stomatologie (chacune 4%) et la neurochirurgie 3%; 29 patients (1.64%) ont présentés un incident et/ou un accident en péri opératoire (Tableau 1); la plupart des effets indésirables sont survenus en per opératoire (58,6%). Dans 28,6% des cas en postopératoire immédiat ou en salle de surveillance post interventionnelle (SSPI). Par ailleurs 4 patients ont présenté des complications quelques heures après la sortie de la SSPI soit 14,8%. La majorité de nos patients ayant présenté un événement indésirable sont des ASA II avec un taux de 59%; la majorité des complications sont survenues au décours d’une anesthésie générale ou une sédation (93%); la plupart des complications survenues sont d’ordre respiratoire (34%) ou cardio vasculaire (31%); les complications enregistrées sont par spécialité et par ordre décroissant: viscérale(31%), ORL(21%), urologie (10%) et traumatologie (10%), plastie (7%), et ophtalmologie (3%), thoracique (3%), gynécologie (3%), neurochirurgie (3%), coloscopie (3%); 62% des patients ayant présentés un événement indésirable ont été transférés à leur service d’origine alors que 38 % ont été transférés en réanimation; 5 patients sont décédés en périopératoire soit une mortalité de 0,28%; le Tableau 2 (suite) illustre l’ensemble des effets indésirables colligés; la détermination de la cause n’est pas toujours évidente. Le facteur humain serait responsable de 24% des complications.

**Tableau 1:** Récapitulatif de l’ensemble des effets indésirables majeurs ou mortels recensés

Incidents	Nb	Descriptions				
		Age	Terrain	Actes	Incidents accidents	Évolution
**Mortels**	5	H60	ASA 2 (valvulaire)	Cystoprostatectomie radicale	Arrêt cardiaque secondaire à une thrombose de la valve mithrale	DCD en réa
		H55	ASA 2 (HTA+ DID)	Anévrysme étendu de l’aorte abdominale	Choc hémorragique réfractaire	DCD en réa
		H63	ASA 2 (HTA+Tabac)	Duodénopancreatectomie céphalique	Retard de réveil avec Arrêt cardiaque 18 h en post opératoire	DCD en réa
		H21	ASA 1	Fracture du coude ALR convertie	Arrêt cardiaque en SSPI (toxicité des anesthésiques locaux?)	DCD en réa
		H65	ASA 2 (HTA+DNID)	Cataracte	14 h en post opératoire IDM compliqué de trouble de rythme	DCD en réa
**Majeurs**	6	F 60	ASA 1	Lithiase vésiculaire	Choc anaphylactique avec bronchospasme	Bonne évolution après 48 H en réa
		F 54	ASA 1	Hystérectomie programmée	Choc hémorragique	Bonne évolution après 12 H en réa
		H55	ASA 1	Septoplastie	Désaturation avec cyanose, défaut du respirateur	Bonne evolution (SSPI puis service)
		F 52	ASA 2 (Valvulopathie)	Greffe de peau	Tachycardie ventriculaire	Bonne évolution après 24 H en réa
		F 64	ASA 2 (DNID)	Tumeur linguale avec reconstruction	Rhabdomyolyse biologique (8h d’intervention)	Bonne évolution après 24h en réanimation
		H58	ASA 2 (HTA+diabète)	Coloscopie	Désaturation en rapport avec une inhalation	Bonne évolution après 24h en réa

**Tableau 2:** Récapitulatif de l’ensemble des effets indésirables mineurs et modérés recensés

Incidents	Nb	Descriptions				
		Age	Terrain	Actes	Incidents accidents	Évolution
**Modéré**	10	H55	ASA 2 (Hémodialysé)	Péritonite perforation ulcère	Thrombose de fistule	Réfection à j 2
		H50	ASA 1	Fracture du poignet, ALR + sédation	Désaturation en post op	Bonne evolution (SSPI puis service)
		F42	ASA 2 (DNID)	Thyroïdectomie	Intubation impossible avec désaturation, ventilation au masque laryngé jusqu’à reprise spontanée, fibroscopie	Bonne evolution (SSPI puis service)
		F20	ASA 1	Cervicotomie	Massage cardiaque et adrénaline à tort (débranchement du scope)	Bonne evolution (Qlq extrasystole)
		F53	ASA 2 (HTA)	Biopsie d’une tumeur cérébrale sous AG	lâchage de la têtière et flexion brutale de la tête	Réveil sans conséquence
		H67	Tabagique chronique	Laryngoscopie directe en suspension	Détresse respiratoire, réintubation et trachéotomie	Bonne evolution (SSPI puis service)
		F24	ASA 1	NLPC	Tachyarythmie supra ventriculaire sans cause évidente	Bonne évolution sous bétabloqueurs
		F54	ASA 1	Tumeur rectale	Brèche durale par obstruction du cathéter par du cartilage	Céphalées intenses traitées blood patch
		H34	ASA 1	Tympanoplastie	Chute en SSPI	Sans conséquence
		F45	ASA 1	Lithiase vésiculaire	Désaturation per op. défaut de manipulation du respirateur	Bonne evolution (SSPI puis service)
**Mineurs**	8	F45	ASA 2 (HTA)	Thyroïdectomie	Difficulté de ventilation, hernie du ballonnet	Bonne évolution après changement de sonde
		H73	ASA 2 (HTA)	Syndrome occlusif	Refus d’intervention. Opéré après aval de la famille	Bonne evolution (bride)
		F43	ASA 1	Lithiase vésiculaire	Éclatement du ballonnet	Bonne évolution après packing
		H53	ASA 2	Thyroïdectomie	Difficulté de ventilation, hernie du ballonnet	Bonne évolution après changement de sonde
		H65	ASA 2 (HTA)	Fracture du col fémur	Après rachianesthésie découverte d’une grosse jambe	Repris après vérification doppler
		H66	ASA 2 (DNID)	Dérivation bilio digestive (tumeur du pancréas)	Convulsion à l’induction au propofol jugulée par du midazolam	Bonne evolution (SSPI puis service)
		F75	ASA 2 (DNID+HTA)	Cholécystite	Bris dentaire (protrusion des incisives + précarité)	Patient compréhensif après explication
		F48	ASA 2 (DNID)	NLPC	Dentier cassé	-

## Discussion

L’amélioration de la sécurité anesthésique passe notamment par une meilleure connaissance des pratiques professionnelles et de leurs conséquences. Pour ce faire, la mise en place d’un observatoire sous la forme d’un système de recueil des actes et des incidents est recommandée par la Société française d’anesthésie et de réanimation, ainsi que par de nombreux experts et sociétés savantes étrangères [2, 3]. Le but de ce recueil systématique est d’obtenir des données épidémiologiques concernant la survenue d’événements indésirables. C’est dans cette perspective que le pôle d’anesthésie réanimation et urgences de notre hôpital a décidé la création d’un registre de morbimortalité. D’autre part, le taux de complications varie d’une étude à l’autre. 23.7% des cas dans une étude française [4, 5], contre 1.64% des cas dans notre étude. Cette différence d’incidence s’explique par la nature de chirurgie, la population étudiée (tares, âge,…..) mais également par les critères de définition retenus pour les accidents collectés. Les complications anesthésiques peuvent survenir à n’importe quel moment durant la période péri opératoire. Pour certains [6] surtout au réveil (42% des cas). Dans notre série plutôt en per opératoire (58,6% des cas). Ces incidents touche essentiellement les sujets ASA I 45% des cas dans une étude française [4]. La même tendance aussi dans notre travail (41%). En fait les sujets ASA II sont toujours mieux surveillés de la part de l’équipe anesthésique.

Les complications respiratoires, comme c’est le cas dans notre travail, sont au premier rang des effets indésirables péri opératoires que ça soit dans les séries nationales [7] qu’internationales [8]. Elles sont fréquentes après chirurgie abdominale [9]. Le même constat a été trouvé dans notre travail, mais doit être nuancé, car plusieurs complications sont sans rapport propre avec ce type de chirurgie. Par ailleurs plusieurs travaux [10, 11] ont montré que le tabac est un facteur de risque de complication péri opératoire (environ 7% dans notre travail). La majorité des complications enregistrées dans notre travail sont au décours d’anesthésie générale (93%). Ce constat est souvent retrouvé dans la littérature [12]. Le manque d’expérience est un facteur de risque de complication durant l’anesthésie [13]. C’est également le cas dans notre série ou le facteur humain est jugé responsable de presque le quart des incidents ce qui souligne l’intérêt d’une formation continue notamment de l’équipe paramédicale. La première lecture de ce registre nous inspire les recommandations suivantes: renforcer l’équipement de surveillance en SSPI ainsi qu’une présence paramédicale continue; assurer une formation continue pour le personnel paramédical; la transmission de consignes entre l’équipe assurant l’anesthésie et celle assurant le réveil doit être systématique et consignée par écrit; l’élaboration de protocole en SSPI permettant le dépistage et la gestion de certains incidents; sans oublier qu’à ces exigences de sécurité, s´ajoutent des objectifs de confort et d´analgésie postopératoire, qui doivent désormais faire partie des standards de soins des patients en SSPI; concernant la chirurgie ambulatoire, aux critères habituels de sortie doivent être associées ceux spécifiquement liés aux conditions propres à notre niveau socio économique.

## Conclusion

L’analyse critique, quoique prématurée, de ce registre de morbidité mortalité a permis de dégager des données épidémiologiques locales, de détecter certains dysfonctionnements et défauts de soins latents, potentiellement source d’accidents, et d’ouvrir des perspectives dans le domaine de l’autoévaluation et de l’enseignement et de mettre en place des actions correctrices qui s’imposent.

### Etat des connaissances actuelleS sur le sujet

- Malgré les importants progrès qui ont été faits dans le domaine de la sécurité en anesthésie, la morbidité liée complètement ou partiellement à l’anesthésie reste cependant fréquente, et aucun praticien n’est aujourd’hui à l’abri d’un accident;
- Dans le contexte actuel où la priorité est donnée à la formation, à l’amélioration de la qualité et de la sécurité des soins, la survenue d’un accident d’anesthésie au bloc opératoire est un événement extrêmement traumatisant;
- L’étude des événements indésirables se cadre dans la revue de morbi-mortalité que chaque établissement de soin doit promouvoir afin d’améliorer la qualité des soins prodigués.

### Contribution de notre étude à la connaissance

- L’analyse critique, quoique prématurée, de notre registre de morbi-mortalité a permis de dégager des données épidémiologiques locales, de détecter certains dysfonctionnements et défauts de soins latents, potentiellement source d’accidents, et d’ouvrir des perspectives dans le domaine de l’autoévaluation et de l’enseignement et de mettre en place des actions préventives et correctrices qui s’imposent;
- Dans notre étude comme dans la littérature le manque d’expérience (facteur humain) est un facteur de risque de complication durant l’anesthésie ce qui souligne l’intérêt d’une formation continue notamment pour l’équipe paramédicale.
